# The Nrf2 Pathway in Liver Diseases

**DOI:** 10.3389/fcell.2022.826204

**Published:** 2022-02-10

**Authors:** Jiaming Zhou, Qiuxian Zheng, Zhi Chen

**Affiliations:** State Key Laboratory for Diagnosis and Treatment of Infectious Diseases, National Clinical Research Center for Infectious Diseases, National Medical Center for Infectious Diseases, Collaborative Innovation Center for Diagnosis and Treatment of Infectious Diseases, The First Affiliated Hospital, Zhejiang University School of Medicine, Hangzhou, China

**Keywords:** reactive oxygen species, nuclear factor-erythroid 2-related factor 2, kelch-like ECH-associated protein 1, oxidative stress, liver diseases

## Abstract

Oxidative stress is the leading cause of most liver diseases, such as drug-induced liver injury, viral hepatitis, and alcoholic hepatitis caused by drugs, viruses, and ethanol. The Kelch-like ECH-associated protein 1-NFE2-related factor 2 (Keap1-Nrf2) system is a critical defense mechanism of cells and organisms in response to oxidative stress. Accelerating studies have clarified that the Keap1-Nrf2 axis are involved in the prevention and attenuation of liver injury. Nrf2 up-regulation could alleviate drug-induced liver injury in mice. Moreover, many natural Nrf2 activators can regulate lipid metabolism and oxidative stress of liver cells to alleviate fatty liver disease in mice. In virus hepatitis, the increased Nrf2 can inhibit hepatitis C viral replication by up-regulating hemeoxygenase-1. In autoimmune liver diseases, the increased Nrf2 is essential for mice to resist liver injury. In liver cirrhosis, the enhanced Nrf2 reduces the activation of hepatic stellate cells by reducing reactive oxygen species levels to prevent liver fibrosis. Nrf2 plays a dual function in liver cancer progression. At present, a Nrf2 agonist has received clinical approval. Therefore, activating the Nrf2 pathway to induce the expression of cytoprotective genes is a potential option for treating liver diseases. In this review, we comprehensively summarized the relationships between oxidative stress and liver injury, and the critical role of the Nrf2 pathway in multiple liver diseases.

## Introduction

The liver is the largest digestive gland in the human body, which accepts dual blood supply of the hepatic artery and portal vein and communicates with the digestive tract through the bile duct. It has essential functions such as secretion of bile, decomposition of sugar and storage of glycogen, detoxification and phagocytosis, and defense. Oxidative stress is a leading cause of liver damage caused by various factors, such as drugs, viruses, ethanol, etc. It will further lead to drug-induced liver injury, fatty liver disease, viral hepatitis, autoimmune liver disease, liver fibrosis, and primary carcinoma of the liver.

The nuclear factor-erythroid 2-related factor 2 (Nrf2) involves multiple aspects of cellular and organismal defense against oxidative stress, such as detoxification, regulating cell metabolism, and promoting cell proliferation, which is crucial in the pathological mechanism of various diseases. Nrf2 is a member of the basic leucine zipper (bZIP) transcription factor in the cap-n-collar (CNC) family. It was first isolated in 1994 as an NF-E2-like bZIP transcription factor, but its function was unclear (Moi et al., 1994). It was found that Nrf2 mediates cytoprotective genes expression through antioxidant responsive elements (ARE) until Nrf2-deficient mice were established and analyzed the stimulation of phase II detoxifying enzymes (Itoh et al., 1997). The expression of cytoprotective genes provides a regulatory network of detoxication enzymes involved in antioxidant metabolism, intermediate metabolism of lipids, protein degradation, and regulation of inflammation. Thus, Nrf2 is able to maintain the steady state of the internal environment responding to diverse forms of stress.

**FIGURE 1:**
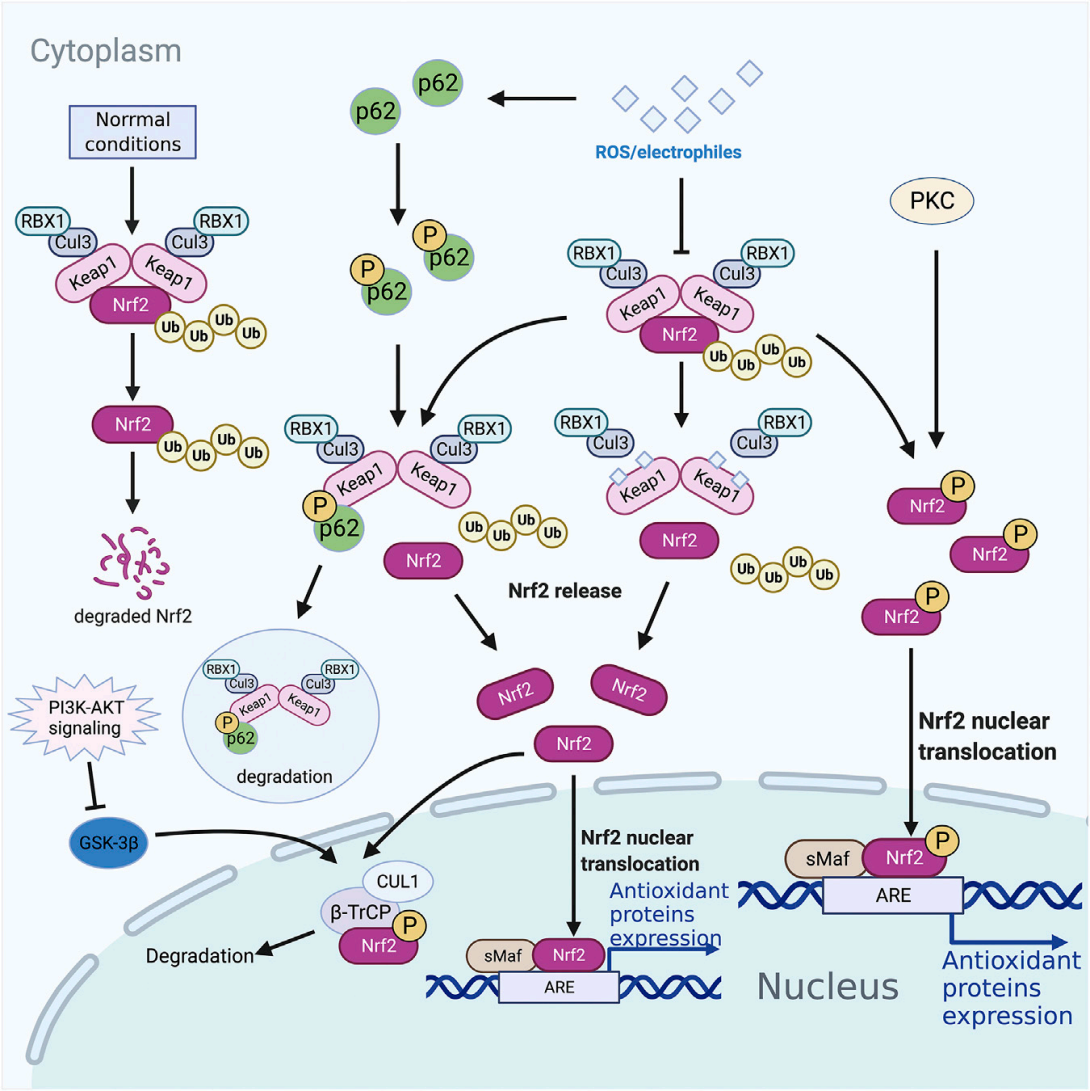
Nrf2 associated pathways. Proteases degrade nrf2 ubiquitinated by the Keap1-CUL3 complex under normal conditions in the cytoplasm. However, under oxidative stress conditions, Nrf2 dissociates from Keap1, accumulates in the cytoplasm, and is transported into the nucleus to bind to target genes. The selective autophagy substrate p62 could compete with Nrf2 for Keap1 binding at the bottom of the DC domain, dislocating Nrf2 from Keap1 and leading to the accumulation of Nrf2, initiating the transcription of antioxidant protective genes and phase II detoxification enzyme genes. PKC phosphorylates Ser40 in Neh2, dissociating the Keap1 homodimer, and transporting Nrf2 into the nucleus to recognize and bind the ARE. Neh6 in Nrf2 can be phosphorylated by GSK-3β, leading to degradation through being recognized by β-TrCP. PI3K-AKT signaling could inhibit GSK-3β through phosphorylation. ARE, antioxidant responsive element; β-TrCP, β-transducin repeats-containing protein; GSK-3, phosphorylated by glycogen synthase kinase 3; Keap1, Kelch-like ECH-associated protein 1; Nrf2, nuclear factor-erythroid 2-related factor 2; ROS, reactive oxygen species; sMAF, small musculoaponeurotic fibrosarcoma oncogene homologue.

Kelch-like ECH-associated protein 1 (Keap1) was originally isolated by Itoh et al. through a yeast two-hybrid system, and it was identified as the primary inhibitor of Nrf2 (Itoh et al., 1999). Mass spectrometry analysis showed that Keap1 was an efficient sensor for redox reactions (Dinkova-Kostova et al., 2002). It was reported to be a substrate adaptor of E3 ubiquitin ligase, which forms a complex with CULLIN3 (CUL3) (Dinkova-Kostova et al., 2002). Under physiological conditions, Keap1 and CUL3 form an E3 ubiquitin ligase complex, and Nrf2 binds to a Keap1 homodimer in the cytoplasm. At this time, the Nrf2 is suppressed in the cytoplasm. When stimulated by reactive oxygen species (ROS) or electrophiles, the hypereactive cysteine residues of Keap1 are modified, and the E3 ubiquitin ligase is inactivated, and the latch is unlocked making the Nrf2 connected to it stable. The newly generated Nrf2 accumulates continuously, and then transports to the nucleus, forms a heterodimer with small musculoaponeurotic fibrosarcoma oncogene homologue (sMAF), recognizes and interacts with the ARE, and initiates the transcription of downstream antioxidant protective genes and phase II detoxification enzyme genes (Figure 1).

It is now widely recognized that Nrf2 act a pivotal part in the ARE-mediated expression of oxidative stress enzyme genes, including antioxidant and detoxicant. Therefore, the Nrf2 pathway is essential to prevent diseases with oxidative stress and inflammation as basic pathologic characters. Oxidative stress is the pathological feature of the most liver diseases. Thus, the Nrf2 pathway may be a potential option for treating liver diseases. We present the current knowledge on the effects of Nrf2 pathway in liver diseases. Furthermore, the mechanism and disputes involved and therapeutic implications are also discussed in this review.

### The Keap1-Nrf2 Pathway

#### Activator of the Keap1-Nrf2 Pathway

ROS is a series of highly reactive substances. Mitochondrial respiratory chain is the main part of ROS production, and enzyme-mediated catalytic reactions such as xanthine oxidase in cytoplasm and cytochrome P450 in microsomes can also produce a small amount of ROS (Turrens, 2003). In addition, bacterial infection, tissue hypoxia, and other pathological processes, drugs, ionizing radiation, and other exogenous factors can also lead to a large number of intracellular ROS (Finkel, 2011). A small amount of ROS acts a pivotal part in biological signals (Finkel, 2011), which can moderate cell proliferation and immune response. However, a large amount of ROS accumulation will damage cell function and even lead to cell death.

#### Keap1-dependent Nrf2 Regulation

**FIGURE 2:**
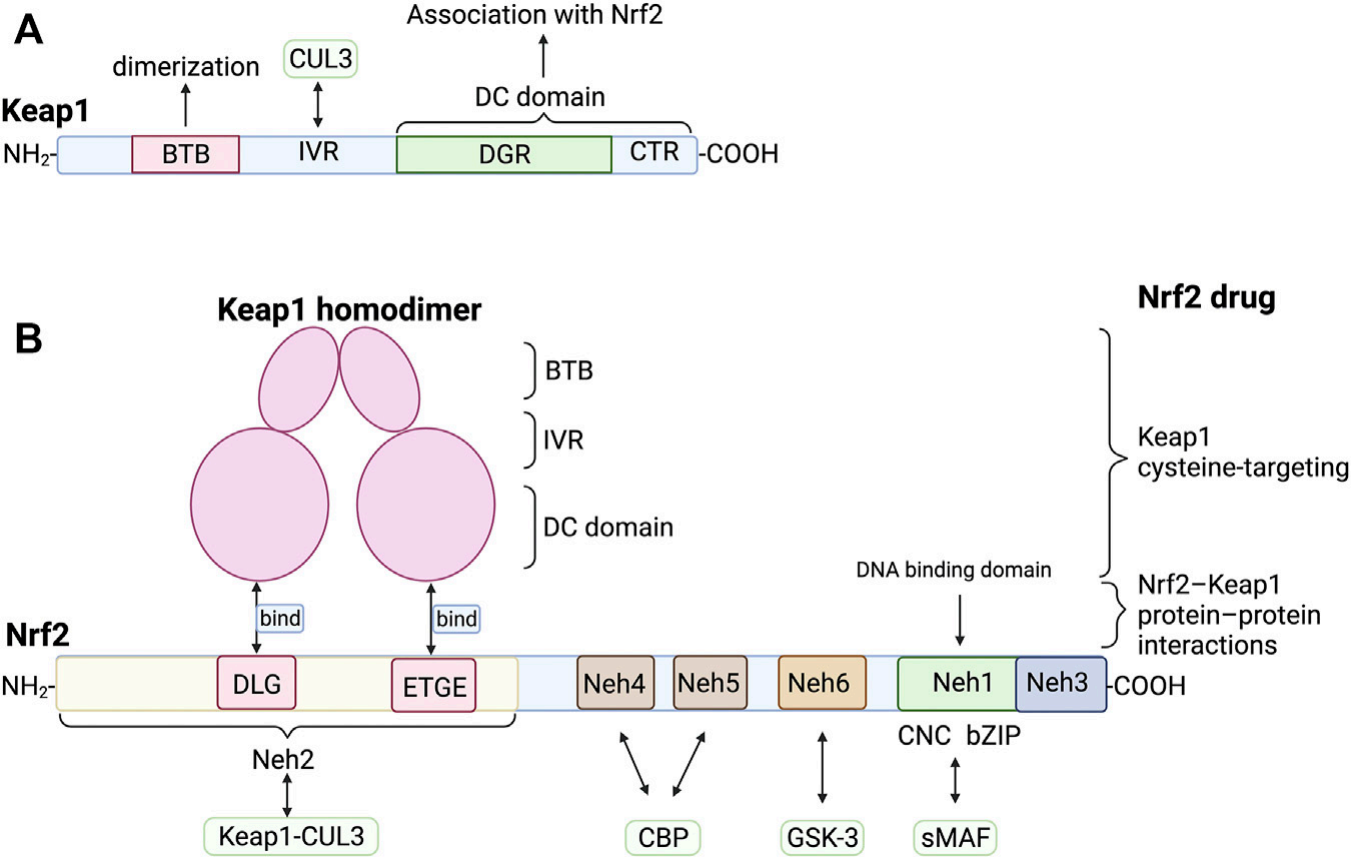
Structure and interaction of Keap1 and Nrf2 **(A)** Keap1 contains BTB domain, IVR domain, DGR domain and CTR domain. Its dimerization is mediated by BTB domain. IVR domain and CUL3 interact to form Keap1-CUL3 complex, which ubiquitinates Nrf2 under normal condition. The DGR and CTR domain are collectively called DC domain, which directly interact with the DLG and ETGE of Neh2 of Nrf2. **(B)** The DLG and ETGE domain on Neh2 could bind with DC domain of Keap1 homodimer. sMAF can bind with Neh1 of Nrf2 to form heterodimers that bind to DNA. Neh6 could be phosphorylated by GSK-3, leading to the degradation of Nrf2. There are several types of Nrf2 activators, such as Keap1 cysteine-targeting drugs and drugs that disrupt the Nrf2-Keap1 protein-protein interactions. BTB, bric-a-brac domain; CBP, cAMP responsive element binding protein; CTR, carboxyl terminal region; CUL3, CULLIN3; DGR, double glycine repeat; GSK-3, glycogen synthase kinase 3; IVR, intermediate region; Neh1, Nrf2-ECH homology domain-1; sMAF, small musculoaponeurotic fibrosarcoma oncogene homologue.

Keap1 was an efficient sensor for redox reaction containing broad complex-tramtrack-bric-a-brac (BTB) domain, intermediate region (IVR), double glycine repeat (DGR), and carboxyl terminal region (CTR). The DGR and CTR domains interact to form a β-helical structure, so these two domains are collectively called the DC domain. Further researches showed that the DC domain of Keap1 could directly interact with the Neh2, carboxyl end of Nrf2 (Figure 2) (Itoh et al., 1999). The BTB is a well-known oligomerization domain. The Keap1 homodimer structure of mice was analyzed by single-particle electron microscopy, revealing that its dimerization was mediated by BTB (Ogura et al., 2010) (Figure 2). The three-dimensional reconstruction showed that two large spheres are connected to the side of a small fork-shaped stem structure by a short connecting arm, similar to a cherry-bob (A pair of cherries with the ends of the rhizomes joined together). Interaction between the IVR domain of Keap1 and the NH2-terminal region of CUL3 forms the Keap1-CUL3 complex (Kobayashi et al., 2004), which can ubiquitinate Nrf2 under normal condition, resulting in degradation of ubiquitinated Nrf2 by proteases. Under the stress of ROS or electrophiles, the hyperreactive cysteine residues such as Cys151 in Keap1 BTB, Cys273 and Cys288 in IVR are mutated (Kobayashi et al., 2006), leading to the inactivation of the E3 ubiquitin ligase of the Keap1-CUL3 complex. The newly synthesized Nrf2 accumulates in the cytoplasm and then transfers to the nucleus to combine with the target gene and induce expression.

#### Nrf2: The Central node of Keap1-Nrf2 Pathway

Nrf2 regulates the induction of the genes related to the generation of NADPH, which is further used by many redox reactions, including malic enzyme 1, 6-phosphogluconate dehydrogenase, isocitrate dehydrogenase 1 and glucose 6-phosphate dehydrogenase (Cuadrado et al., 2019). Thus, through the induction of key enzymes related to the synthesis and consumption of the reduced glutathione (GSH), many redox reactions are regulated by Nrf2. At the same time, Nrf2 regulates genes encoding the most effective physiological antioxidant enzymes, such as hemeoxygenase-1 (HO-1), biliverdin reductase (BVR), and NAD(P)H: quinone oxidoreductase 1 (NQO1).

Nrf2 contains six highly conserved homology domains which are termed Neh1 (Nrf2-ECH homology domain-1) ∼ Neh6 (Figure 2). The CNC and bZIP domains are located in Neh1, and the amino end and carboxyl end are called Neh2 and Neh3. Moreover, Itoh et al. further found that the Neh2 domain deleted Nrf2 mutant showed higher transcriptional activity, indicating that the Neh2 domain was involved in the negative regulation of Nrf2. (Itoh et al., 1999). DLG and ETGE motifs in Neh2 are necessary for its interaction with Keap1 (Kobayashi et al., 2002; Katoh et al., 2005). Isothermal calorimetry analysis showed that a Neh2 molecule interacted with two Keap1 molecules through two binding sites, namely the ETGE motif with stronger binding force and the DLG motif with weaker binding force (Tong et al., 2006). Nuclear magnetic resonance titration studies have shown that ETGE and DLG bind to overlapping sites on the bottom surface of the beta-propeller structure of Keap1 (Tong et al., 2006). Neh4 and Neh5 are acidic conserved sequences that enhance the expression of Nrf2 target genes by binding cAMP responsive element binding protein (Katoh et al., 2001). Neh6 is a serine-rich conserved sequence, which promotes the degradation of Nrf2 by being phosphorylated by glycogen synthase kinase 3 (GSK-3) (Salazar et al., 2006).

As mentioned above, Nrf2 ubiquitinated by the Keap1-CUL3 complex under normal conditions is degraded by proteases in the cytoplasm. However, under oxidative stress conditions, Nrf2 dissociates from Keap1, accumulates in the cytoplasm, and is transported into the nucleus to bind to target genes.

#### sMAF: A Partner of Nrf2

sMAF is a bZIP transcription factor in the MAF family, which mainly exists in MAFF, MAFG, and MAFK in mammals (Yamamoto et al., 2018). A significant role of sMAF is to combine with CNC proteins to form heterodimers, allowing them to bind to the ARE motif on DNA, thereby exerting transcriptional regulation (Katoh et al., 2001). Katsuoka F et al. found that the induction of the ARE-dependent gene was severely impaired in sMAF deficient mice, further confirming this view (Katsuoka et al., 2005). More and more studies have confirmed that: Nrf2-sMAF heterodimer is a trans-acting factor that can recognize ARE and initiates the transcription of downstream antioxidant protective genes and phase II detoxification enzyme genes.

#### Target Genes: Cell Protection and Detoxification

In 1990, Rushmore et al. found a new biological element that can directly react with phenolic antioxidants (such as t-butylhydroquinone) in mice, and named it as antioxidant response element, also known as electrophile response element (EpRE) (Rushmore and Pickett, 1990). The ARE activation can initiate the transcription of downstream antioxidant protective genes and phase II detoxification enzyme genes, which translates into HO-1, superoxide dismutase (SOD), NQO1, catalase, glutathione peroxidase, glutamate-cysteine ligase, glutathione-S-transferase and epoxide hydrolase, etc.

### Keap1-Nrf2 Pathway and Autophagy

The selective autophagy substrate p62 contains an ETGE-like motif, STGE, which could be phosphorylated by mTOR complex 1 to bind to Keap1 with higher affinity (Ichimura et al., 2013). At the same time, Keap1 bound to p62 is degraded in autophagosomes (Taguchi et al., 2012). Thus, p62 competes with Nrf2 for Keap1 binding at the bottom of the DC domain, dislocating Nrf2 from Keap1 and leading to the accumulation of Nrf2 (Figure 1) (Komatsu et al., 2010). There is a positive feedback loop between p62 and Nrf2: p62 is a target gene for Nrf2, promoting Nrf2 stimulation and Nrf2 promotes p62 production (Jain et al., 2010).

### Keap1-independent Mechanism of Nrf2 Regulation

Although the majority of Nrf2 is mainly moderated by Keap1 in answer to ROS/electrophiles, some researchers found that the PI3K-AKT signaling pathway could also stimulate Nrf2 (Figure 1) (Martin et al., 2004; Li et al., 2006). GSK-3β is a key mediator of PI3K-AKT-Nrf2 signaling pathway, and it can be suppressed by AKT-mediated phosphorylation (Salazar et al., 2006). Neh6 in Nrf2 contains serine residues, which can be phosphorylated by GSK-3β so that Nrf2 can be recognized by β-transducin repeats-containing protein (β-TrCP) and be recognized by the ubiquitin ligase of β-TrCP-CUL1 complex degradation (Rada et al., 2012; Chowdhry et al., 2013).

Oxidative stress-induced PKC can catalyze the serine and threonine residues on Ser40 in Neh2, dissociating the Keap1 homodimer, and transporting Nrf2 into the nucleus to recognize and bind the ARE/EpRE response element (Figure 1) (Huang et al., 2002; Fei et al., 2017). Of note, *in vitro* studies have shown that regardless of the phosphorylation status of Nrf2, the affinity of Nrf2-sMaf heterodimers to ARE is similar (Kensler et al., 2007).

### Development of Nrf2 Inducers

For exploring the preventive and alleviating efforts of Nrf2 in mouse disease models, the whole world is striving to isolate or develop effective and potentially specific Nrf2 inducing chemicals from natural sources. Nrf2 agonists that have made progress in mouse disease models: Oleanolic acid derivatives CDDO-Me and CDDO-Im, 15d-PGJ_2_ [15-deoxy-Delta (12,14)-prostaglandin J (2)], oltipraz, fumarate, triterpenoids (such as bardoxolone methyl, BARD) and sulforaphane (SFN), etc (Gong et al., 2002; Yamamoto et al., 2018; Cuadrado et al., 2019). These agonists can initiate the Nrf2-ARE pathway and downstream target genes, thereby reducing or preventing the occurrence and development of oxidative stress. Dimethyl fumarate (DMF) is the only Nrf2 agonist approved by the Food and Drug Administration (FDA) and the European Drug Administration (EMA) for the treatment of multiple sclerosis and psoriasis (Linker et al., 2011; Brück et al., 2018). DMF is metabolized into MMF (monomethyl fumarate) in the body. MMF inactivates Keap1 by forming an adduct at C151, thereby activating Nrf2. Some biomedical companies are beginning to develop MMF compounds, which compared with DMF have higher bioavailability and fewer side effects. In addition, Japan initiated a phase III clinical trial of BARD for diabetes mellitus patients with CKD in the G3 or G4 stage (Cuadrado et al., 2019). Broccoli sprout (BS) is a dietary supplement riching in sulforaphane. In a randomized, placebo-controlled, double-blind trial, male subjects with hepatic abnormalities and who were diagnosed with fatty liver using ultrasonography were treated with BS capsules or placebo for 2 month. It found that BS significantly decreased serum levels of liver function markers after 2 month (Kikuchi et al., 2015).

### Drug-Induced Liver Injury (DILI)

**FIGURE 3:**
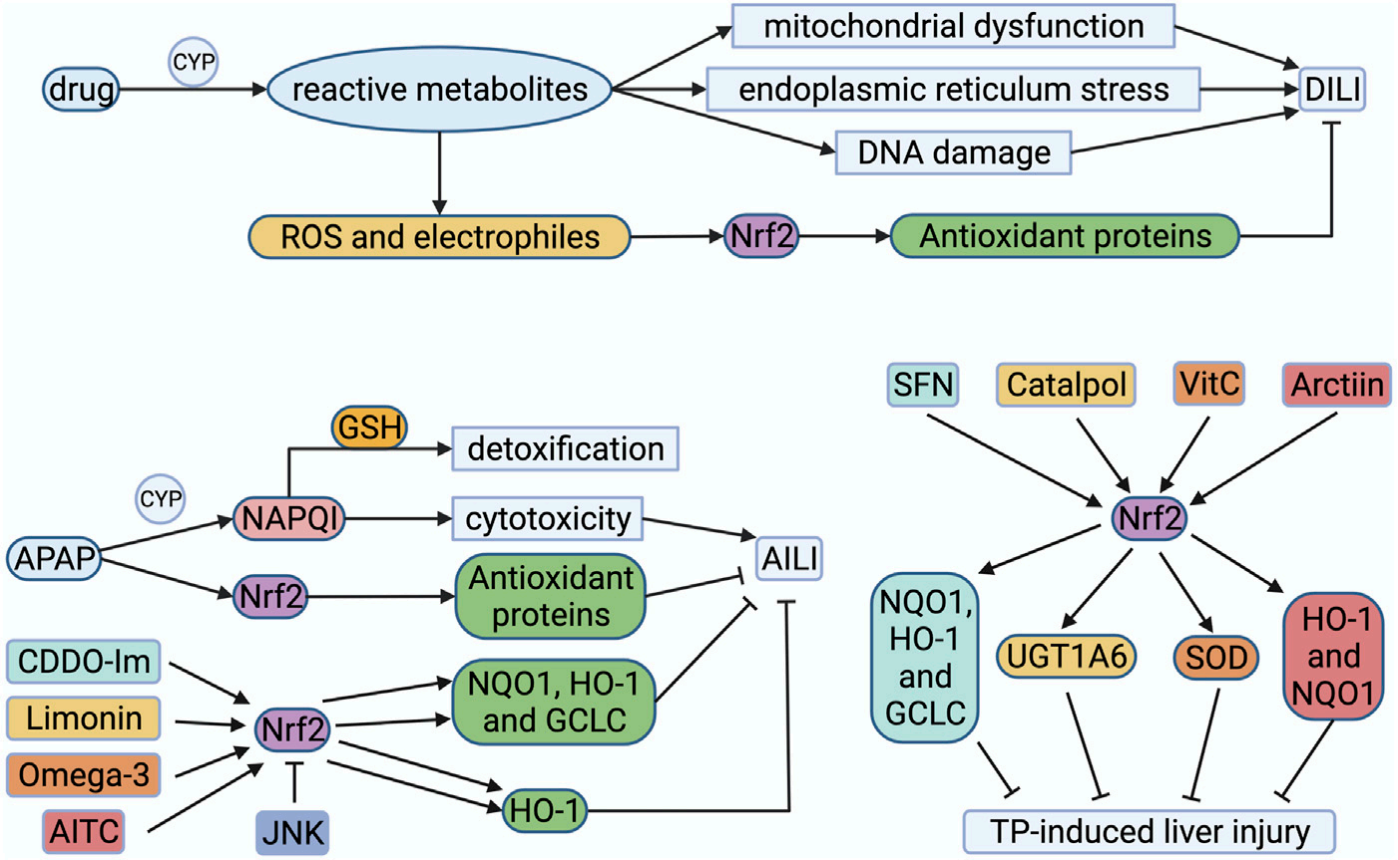
Drug-induced liver injury and Nrf2 drugs. Drugs metabolized by CYP are more likely to cause DILI. Reactive metabolites give rise to mitochondrial dysfunction, endoplasmic reticulum (ER) stress, or DNA damage. ROS and electrophiles activate Nrf2 to compound antioxidant proteins, alleviating DILI. For example, APAP can be metabolized by CYP to produce NAPQI, which detoxified by GSH at first. As APAP dosage increases, GSH exhausts and NAPQI accumulates, resulting in cytotoxicity. Reactive metabolites like ROS could stimulate Nrf2, initiating the transcription of target genes. CDDO and limonin could activate Nrf2 to compound NQO1, HO-1 and GCLC, which alleviate AILI. Omega-3 and AITC could also stimulate Nrf2 to compound HO-1 for alleviating AILI. But JNK phosphorylates Nrf2 to downregulate the transcriptional expression of cytoprotective genes in AILI. SFN, catalpol, Vitamin C and arctiin alleviate the liver damage induced by triptolide by activating Nrf2 to compound corresponding antioxidant proteins. AITC, allyl isothiocyanate; APAP, acetaminophen; CYP, cytochrome P450 proteins; DILI, drug-induced liver injury; GCLC, catalytic subunit of glutamate-cysteine ligase; GSH, reduced glutathione; HO-1, hemeoxygenase-1; JNK, c-Jun NH2 -terminal kinase; NAPQI, N-acetyl-1,4-benzoquinone imine; NQO1, quinone oxidoreductase 1; Nrf2, nuclear factor-erythroid 2-related factor 2; ROS, reactive oxygen species; SOD, superoxide dismutase; UGT1A6, UDP-glucuronosyltransferase 1A6.

Liver is the main target organ of drug toxicity, because it acts a crucial part in human drug metabolism and clearance. DILI refers to adverse reactions to drugs or exogenous substances. It is often used to describe the accidental damage that drugs may cause to the liver (hepatocytes and other liver cells), such as acetaminophen (APAP) and isoniazid and statins and so on. The US FDA has studied the data of 254 medicines in the LTKB-BD (Liver Toxicity Knowledge base Benchmark Dataset) and found that regardless of the administered dose, drugs metabolized by cytochrome P450 (CYP) enzymes CYP1A2, CYP2C8/CYP2C9 and CYP3A5 were more likely to cause DILI (Yu et al., 2014). Reactive metabolites (such as ROS and electrophiles) can promote adaptive responses through mitochondrial dysfunction, endoplasmic reticulum stress, or DNA damage, thereby increasing molecular chaperone proteins (Figure 3). These molecular chaperone proteins can prevent misfolding in organelles or activate gene regulatory elements through redox-activated transcription factors (such as Nrf2) to protect cells (Andrade et al., 2019).

APAP is widely used as an antipyretic and analgesic. Overdose of APAP is a major cause of acute liver failure, and is also a classic model of DILI. APAP is an oral drug absorbed through the intestine and metabolized by the liver. When APAP is taken in small doses, most drugs are cleared by sulfation and glucuronidation, and only a tiny amount of drugs can be metabolized by cytochrome P450 enzymes to produce N-acetyl-1,4-benzoquinone imine (NAPQI), which is detoxified by binding to GSH in the liver (Kaplowitz et al., 2015). As the dosage of APAP increases, the concentration of APAP in liver cells increases. Most APAP is metabolized by the P450 enzyme CYP2E1 to generate NAPQI and combine with GSH to detoxify (van Rongen et al., 2016). However, with the depletion of GSH in liver cells, reactive metabolites gradually accumulate, resulting in cytotoxicity, which eventually leads to liver cell necrosis and acute liver failure (Figure 3). APAP can produce ROS during liver injury, thereby inducing the activation of Nrf2 (Enomoto et al., 2001). Therefore, Nrf2 could be expected to treat APAP-induced liver injury (AILI). Some researchers found that Nrf2-knockout mice are more sensitive to APAP than wild-type mice (Chan et al., 2001). When a dose of APAP tolerated by wild-type mice was injected intraperitoneally, Nrf2-knockout mice died of acute liver failure. CDDO-Im is a triterpenoid, which can induce the transcription and expression of cytoprotective genes via the Keap1-Nrf2 signaling pathway. Investigators pretreated wild-type mice with CDDO-Im, and they could observe an increase in Nrf2 protein expression, which induced downstream NQO1, HO-1 and GCLC gene expression, and alleviated liver damage caused by APAP (Figure 3) (Reisman et al., 2009). However, the researchers did not observe the protective effect of CDDO-Im on the liver in Nrf2 knockout mice (Reisman et al., 2009). In recent years, people have actively explored the therapeutic applications of natural products with antioxidant activity on AILI, such as limonin, omega-3 fatty acids, allyl isothiocyanate, etc., which can activate the Nrf2 signaling pathway (Yang et al., 2020a; Eraky and Abo El-Magd, 2020; Kim et al., 2020). In 2020, several investigators discovered that JNK (c-Jun NH2 -terminal kinase) phosphorylates Nrf2 Neh6 to downregulate the transcriptional expression of cytoprotective genes in AILI (Chen et al., 2020). It puts forward new ideas for the treatment of AILI.

In a nationwide retrospective study, it was estimated that the annual incidence of DILI in China is 23.80 cases per 100,000, which is much higher than Western countries. Traditional Chinese medicines (TCM) or herbal and dietary supplements (26.81%) and antituberculosis medications (21.99%) are the main causes of DILI in mainland China (Shen et al., 2019a). Triptolide (TP) is a diterpenoid extracted from the TCM Tripterygium wilfordii, which has anti-leukemia and anti-tumor activities. The metabolism of TP is mainly catalyzed by the hepatic cytochrome P450 enzyme. Researchers found that TP can deplete GSH in liver cells and increase ROS, which induces oxidative stress (Li et al., 2014). At the same time, they used sulforaphane (a classic Nrf2 agonist) to treat BALB/C mice treated with TP and found that sulforaphane could alleviate TP-induced liver injury, GSH and antioxidant enzyme consumption (Li et al., 2014). Therefore, activation of Nrf2 can prevent TP-induced liver injury. In recent years, a large number of studies have shown that natural Nrf2 agonists such as catalpol, vitamin C, and arctiin can alleviate the liver damage induced by triptolide by activating Nrf2 (Figure 3) (Xu et al., 2019; Fu et al., 2020; Zhou et al., 2020).

### Fatty Liver Disease

#### Non-alcoholic Fatty Liver Disease (NAFLD)

**FIGURE 4:**
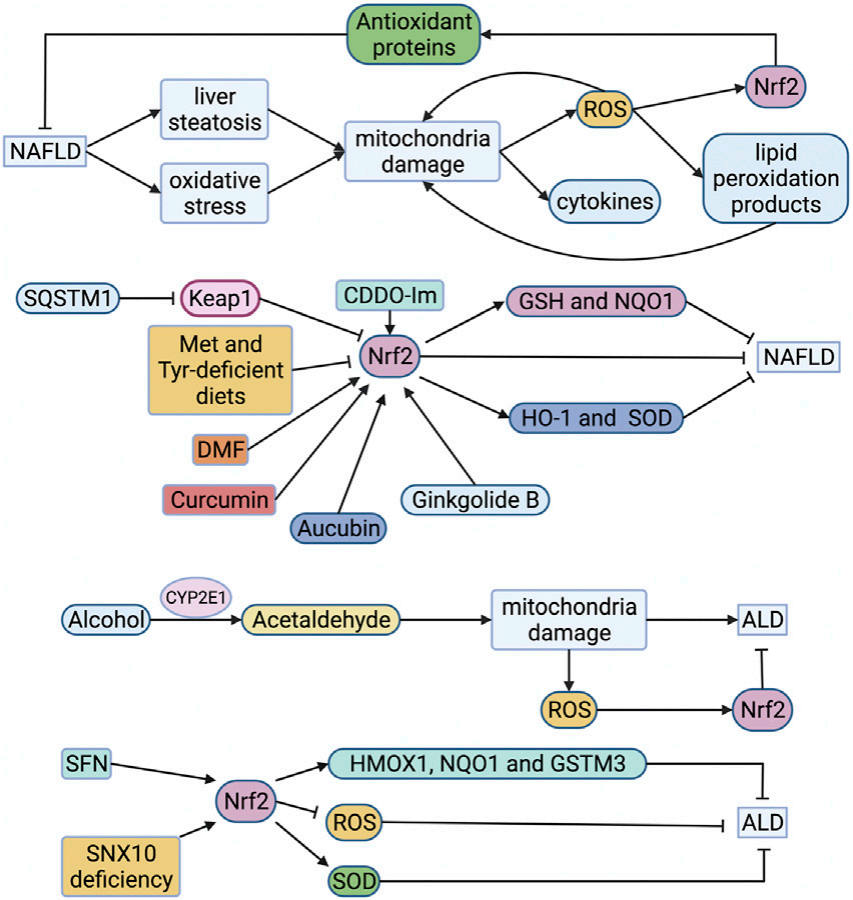
Roles of Nrf2 in FLD. Mitochondria in NAFLD is impaired, leading to overproduction of ROS which triggers lipid peroxidation. The generated ROS and lipid peroxidation products will further damage the function of the respiratory chain, resulting in a vicious circle. ROS also activates Nrf2 to compound antioxidant proteins, alleviating NAFLD. Expression of Nrf2 is much higher in the livers of mice which fed with high-fat diet than those fed with normal chow. However, the accumulation of Nrf2 is inhibited when the intake of Met and Tyr is restricted in high-fat feeding mice. CDDO-Im, DMF, curcumin and ginkgolide B can alleviate NAFLD through activating Nrf2. Aucubin stimulates Nrf2 to compound HO-1 and SOD, alleviating NAFLD. Alcohol is metabolized to acetaldehyde in liver cells by alcohol dehydrogenase and CYP2E1. Acetaldehyde can destroy mitochondria, resulting in the production of ROS. ROS activates Nrf2 to alleviate ALD. SFN stimulates Nrf2 to compound HMOX1, NQO1 and GSTM3 for alleviating NAFLD. The up-regulation of Nrf2 was observed in knockout SNX10 mice. And SOD was compounded to alleviate ALD. ALD, alcoholic liver disease; CYP2E1, cytochrome P450 2E1; DMF, dimethyl fumarate; GSH, reduced glutathione; GSTM3, glutathione S-transferase 3; HMOX1, heme oxygenase 1; HO-1, hemeoxygenase-1; Keap1, Kelch-like ECH-associated protein 1; Met, methionine; NAFLD, non-alcoholic fatty liver disease; NQO1, quinone oxidoreductase 1; Nrf2, nuclear factor-erythroid 2-related factor 2; ROS, reactive oxygen species; SFN, sulforaphane; SNX10, sorting nexin 10; SOD, superoxide dismutase; SQSTM1, sequestosome 1; Tyr, tyrosine.

NAFLD is a lipotoxic disease characterized by liver steatosis and oxidative stress. Mitochondria show many structural and functional abnormalities in ianimal models and non-alcoholic steatohepatitis (NASH) patients (Sunny et al., 2017). The impaired function of the mitochondrial respiratory chain leads to overproduction of ROS and cytokines, which triggers lipid peroxidation. Moreover, the generated ROS and lipid peroxidation products will further damage the function of the respiratory chain, resulting in a vicious circle (Figure 4) (Begriche et al., 2006). The increased expression and activity of CYP2E1 in NAFLD is also one of the critical reasons for the production of large amounts of ROS. In a cohort study of 63 patients with NAFLD, immunohistochemical analysis of pathological liver tissues showed that patients with chronic liver diseases had increased oxidative stress and high Nrf2 expression (Mohs et al., 2021). In the children’s NAFLD cohort, RNA sequencing of pathological liver tissues showed that Nrf2 activation was related to the degree of inflammation, but not to the level of steatosis (Mohs et al., 2021). This conclusion was confirmed in the adult NASH cohort. In the mouse experiment of this cohort study, the investigators found that knocking out Keap1 resulted in the expression of Nrf2 target genes (such as GSH and NQO1), which supplemented the consumption of GSH in the oxidative stress response (Mohs et al., 2021). Shin et al. found that CDDO-Im treatment of high-fat diet-induced obesity in the wild-type mice can prevent lipid accumulation, but this phenomenon did not occur in Nrf2-knockout mice (Figure 4) (Shin et al., 2009). It also found that expression of Nrf2 was much higher in the livers of mice which fed with a high-fat diet than those fed with normal chow (Sano et al., 2021). Although the NAFLD-like phenotype was observed in mice fed with high-fat-Met and Tyr-deficient diets, Nrf2 accumulation was suppressed where nuclear transport of Nrf2 and fumarate in the liver was reduced (Sano et al., 2021). In contrast, mice that knocked out Keap1 ameliorated this phenomenon. In addition, dimethyl fumarate ameliorated the steatosis and increased the hepatic fumarate that was minified due to the loss of Met and Tyr *in vitro* (Sano et al., 2021). Some scholars have discovered by knocking out the mouse SQSMT1 gene that SQSTM1 mediates ULK1 phosphorylation to activate autophagy and promote the formation of AMPK-ULK1-SQSTM1 complex, which leads to autophagic Keap1 degradation and activates the non-canonical Keap1-Nrf2 pathway, thereby protecting mouse liver from lipotoxicity (Figure 4) (Lee et al., 2020). Moreover, many natural Nrf2 activators such as curcumin, aucubin and ginkgolide B can alleviate NAFLD through the regulation of lipid metabolism and oxidative stress of liver cells, which may be a new prevention and treatment for NAFLD (Figure 4) (Yan et al., 2018a; Shen et al., 2019b; Yang et al., 2020b).

#### Alcoholic Liver Disease (ALD)

ALD is mainly caused by heavy drinking. It can gradually evolve from alcoholic fatty liver to alcoholic steatohepatitis (ASH). Chronic ASH can evolve into liver fibrosis, cirrhosis, and even hepatocellular carcinoma (HCC). Alcohol is metabolized to acetaldehyde in liver cells by alcohol dehydrogenase and cytochrome P450 2E1 (CYP2E1) (Lieber et al., 1970). Acetaldehyde can change the structure of mitochondria and cause its dysfunction, including damage to the respiratory chain, resulting in a decrease in ATP, the production of ROS, and a decrease in the activity of acetaldehyde dehydrogenase (Seitz et al., 2018). In addition, CYP2E1 has high NADPH oxidase activity, which can induce NADH transport to mitochondria and increase the ROS production (Bailey and Cunningham, 2002). Researchers evaluated ethanol-induced chronic liver injury, steatosis, and oxidative stress in mice. They found that ethanol caused fatty liver and oxidative stress in wild-type mice, attenuated in CYP2E1 knockout mice, but recovered in humanized CYP2E1 knock-in mice (Lu et al., 2010). This indicates that CYP2E1 acts a crucial part in ethanol-induced fatty liver and oxidative stress (Figure 4). In the cytological experiment of exposure to ethanol, it was found that Nrf2 was up-regulated as an adaptive response to CYP2E1-mediated oxidative stress (Seitz et al., 2018). Therefore, Nrf2 can be a potential therapeutic target for ALD. Ishida et al. found that sulforaphane can inhibit the proliferation and fibrosis of LX-2 cells induced by acetaldehyde by up-regulating Nrf2-regulated antioxidant genes (HMOX1, NQO1 and GSTM3), and reducing lipid accumulation and peroxidation (Figure 4) (Ishida et al., 2021). You et al. found that the lack of SNX10 (Sorting nexin 10) inhibits the maturation of cathepsin A (CTSA) and increases the stability of lysosome-associated membrane protein type 2A (LAMP-2A), leading to chaperone-mediated autophagy (CMA) activation (Figure 4) (You et al., 2018). They further interfered with LAMP-2A and found that the up-regulation of Nrf2 in knockout SNX10 mice depends on CMA activation, thereby reducing alcohol-induced liver damage and steatosis (You et al., 2018). It may become a potential therapeutic target.

### Viral Hepatitis

**FIGURE 5:**
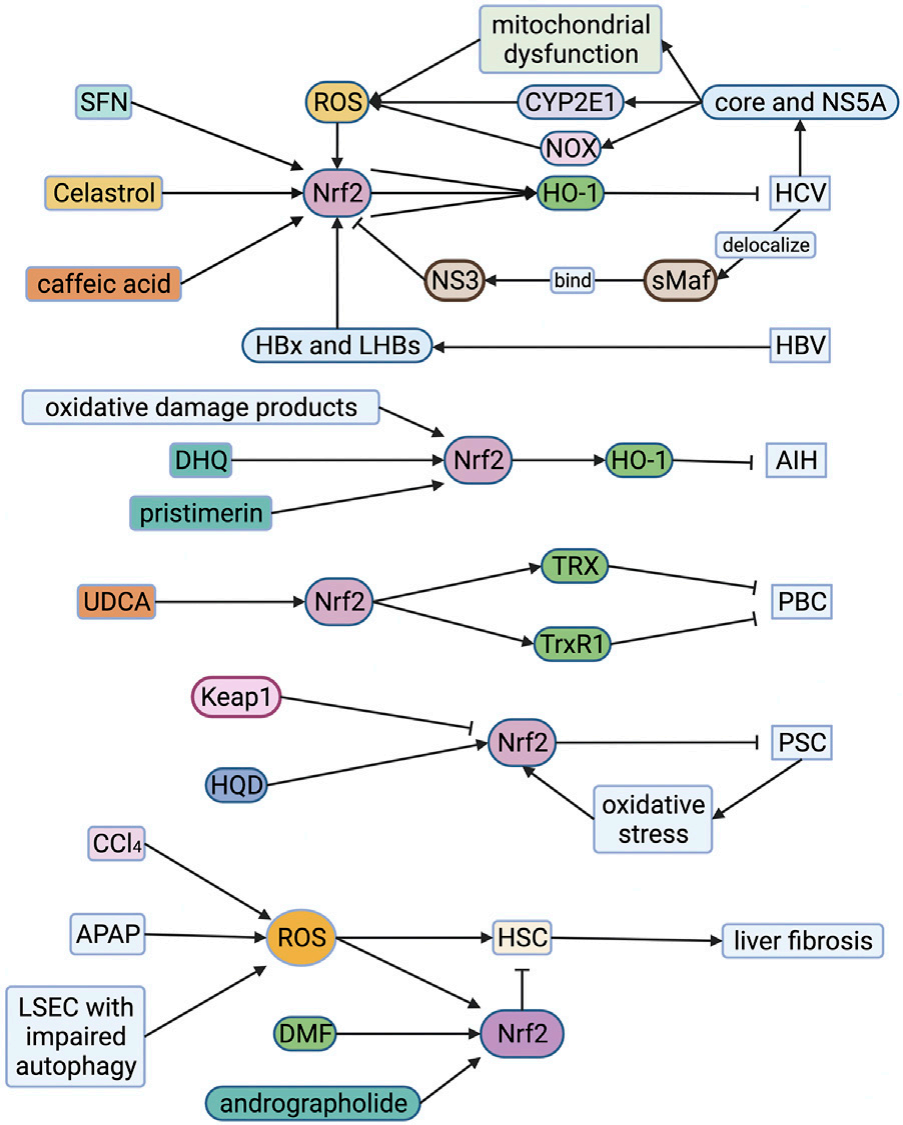
Roles of Nrf2 in viral hepatitis, autoimmune liver disease and liver fibrosis. HCV core protein and NS5A protein induce mitochondrial dysfunction, CYP2E1 and NOX expression in hepatocytes producing large amounts of ROS. HCV core protein and NS5A protein also induce Nrf2 phosphorylation, resulting in up-regulation of HO-1 and NQO1 which alleviate HCV. However, HCV could cause sMAF to delocalize and connect with extranuclear NS3, and then bind to Nrf2 in the cytoplasm, preventing Nrf2 from entering the nucleus. SFN, celastrol and caffeic acid can up-regulate the expression of HO-1 through the Nrf2-associated pathway to inhibit HCV viral replication. The HBx protein of HBV can induce intense stimulation of Nrf2. At the same time, HBV enhances the reciprocity between p62 and Keap1, forming a HBx-p62-Keap1 complex in the cytoplasm, prompting the dissociation of Keap1-Nrf2, which contributes to activation of Nrf2. Oxidative damage products, dihydroquercetin (DHQ) and pristimerin increase the expression of Nrf2 in the cytoplasm, significantly enhancing the transcriptional expression of the HO-1 alleviating AIH. Ursodeoxycholic acid (UDCA) enhances the activation of Nrf2 in liver cells of PBC patients, increasing TRX and TrxR1 protein which alleviate PBC. Huangqi Decoction (HQD) up-regulates the expression of Nrf2 for alleviating PSC. ROS is one of the activating factors of hepatic stellate cells (HSC). It also promotes the activation of Nrf2 which suppresses HSC. Andrographolide and dimethyl fumarate (DMF) can significantly ameliorate the stimulation of HSC by enhancing Nrf2 and increasing the expression of antioxidant proteins. The damage of sinusoidal endothelial cells (LSEC) during acute liver injury can aggravate the oxidative stress response and activate HSC to promote liver fibrosis. The increase of p62 level caused by impaired autophagy will trigger the stimulation of Nrf2 and the up-regulation of its target gene, alleviating liver fibrosis. AIH, autoimmune hepatitis; APAP, acetaminophen; CCl_4_, carbon tetrachloride; CYP2E1, cytochrome P450 2E1; DHQ, dihydroquercetin; DMF, dimethyl fumarate; HO-1, hemeoxygenase-1; HQD, Huangqi Decoction; HSC, hepatic stellate cell; Keap1, Kelch-like ECH-associated protein 1; LSEC, liver sinusoidal endothelial cell; Nrf2, nuclear factor-erythroid 2-related factor 2; NOX, NADPH oxidase; PBC, primary biliary cholangitis; PSC, primary sclerosing cholangitis; ROS, reactive oxygen species; SFN, sulforaphane; sMAF, small musculoaponeurotic fibrosarcoma oncogene homologue; TRX, thioredoxin; TrxR1, thioredoxin reductase 1; UDCA, ursodeoxycholic acid.

The aerobic metabolism of mitochondria and oxidative stress prompt the occurrence and development of chronic HBV and HCV (Mansouri et al., 2018). HCV core protein and NS5A protein are considered to be the main activators that induce mitochondrial dysfunction, CYP2E1 and NADPH oxidase expression in hepatocytes producing large amounts of ROS and lipid peroxides (Figure 5) (Otani et al., 2005; Ivanov et al., 2015; Smirnova et al., 2016). Due to ROS accumulation, HCV core protein and NS5A induce Nrf2 phosphorylation through the PKC pathway. At the same time, they can also induce Nrf2 phosphorylation through the ROS-independent CK2 and PI3K pathways, thereby up-regulating the expression of HO-1 and NQO1 (Ivanov et al., 2011). However, HCV could cause sMAF to delocalize and connect with extranuclear NS3, and then bind to Nrf2 in the cytoplasm, preventing Nrf2 from entering the nucleus binding to ARE, inhibiting target genes expression (Carvajal-Yepes et al., 2011). But the induction of Keap1-Nrf2 signaling pathway is still a potential therapeutic target of HCV. Yu et al. found that sulforaphane can up-regulate the expression of HO-1 through the PI3K/Nrf2 signaling pathway to inhibit HCV virus replication (Yu et al., 2016). Celastrol can also up-regulate HO-1 through the JNK MAPK/Nrf2 signaling pathway to inhibit HCV viral replication (Tseng et al., 2017). In addition, caffeic acid inhibits HCV replication through the p62-mediated Keap1-Nrf2 signaling pathway induced HO-1 expression and IFNα antiviral response (Figure 5) (Shen et al., 2018).

The HBx protein of HBV is considered to be an essential cause of mitochondrial dysfunction. It can change various mitochondrial-related functions, including increasing the production of mitochondrial ROS, reducing mitochondrial transmembrane potential, and changing mitochondrial calcium homeostasis (Mansouri et al., 2018). HBV regulatory proteins (HBx and LHBs) can induce intense stimulation of Nrf2/ARE regulatory genes *in vivo* and *in vitro* through c-Raf and MEK (Figure 5) (Schaedler et al., 2010). In addition, the researchers found that HBV enhances the reciprocity between p62 and Keap1 through binding HBx to p62, forming a HBx-p62-Keap1 complex in the cytoplasm, prompting the dissociation of Keap1-Nrf2, which contributes to activation of Nrf2 and up-regulation of G6PD expression. It shows that HBV can affect glucose metabolism in liver cells, which may be potentially connected with the development of HBV-related liver cancer (Liu et al., 2015). Therefore, on the one hand, Nrf2 is a protective factor for viral hepatitis, and on the other hand, it may contribute to the occurrence and progress of liver cancer.

### Autoimmune Liver Disease

#### Autoimmune Hepatitis (AIH)

Regardless of the cause, ROS can be released and accumulated from Kupffer cells and liver cells during liver inflammation (Czaja, 2011). Researchers analyzed the oxidative damage products and antioxidant components in the serum and urine of 36 AIH patients. They found that compared to healthy subjects, the levels of lipid and protein oxidative damage products in AIH patients were increased, and the glutathione levels were significantly reduced (Figure 5) (Kaffe et al., 2015). The Nrf2-HO-1-CO pathway can regulate immune inflammatory response by up-regulating antioxidant activity and metabolites (such as CO), and is an enzymatic pathway to suppress immune-related inflammation (Ryter and Choi, 2016). The mouse hepatitis model induced by intravenous concanavalin A (ConA) is a classic model of AIH. Zhao et al. found that dihydroquercetin increased the expression of Nrf2 in the cytoplasm, significantly enhancing the transcriptional expression of the HO-1, and alleviating ConA-induced liver damage in mice (Zhao et al., 2015). El-Agamy et al. found that pristimerin can enhance the expression and binding ability of Nrf2, increase the transcriptional expression of HO-1, and have a protective effect on ConA-induced hepatitis (Figure 5) (El-Agamy et al., 2018). Thus, Nrf2 may become a potential therapeutic target.

#### Primary Biliary Cholangitis (PBC)

PBC, also known as primary biliary cirrhosis, is a chronic cholestatic disease caused by chronic progressive non-purulent inflammation of the small bile ducts in the liver. In order to find out the cytoprotective mechanisms of ursodeoxycholic acid (UDCA) in PBC, Kawata et al. used liver biopsy to compare the liver cell status and Nrf2-mediated antioxidant protein of 13 PBC patients before and after UDCA treatment and collected serological samples for analysis such as serum total bilirubin, alkaline phosphate Enzymes and other indicators. They found that the activation of Nrf2 in liver cells of PBC patients was enhanced after UDCA treatment and thioredoxin (TRX) and thioredoxin reductase 1 (TrxR1) protein significantly increased (Figure 5) (Kawata et al., 2010). In addition, Motteleb et al. observed an increase in the induction of Nrf2 and its downstream genes such as HO-1, GSH, and SOD in preventing liver fibrosis caused by bile duct ligation in rats with sildenafil (Shearn et al., 2019). Thus, Nrf2 may become a potential therapeutic target.

#### Primary Sclerosing Cholangitis (PSC)

Shearn et al. used multi-drug resistance protein 2 knockout (MDR2-KO) mice as PSC models and analyzed liver tissues and hepatocyte extracts from wild-type mice. They found that cholestasis induces oxidative stress around the portal vein and increases the production of reactive aldehydes (Shearn et al., 2019). Li et al. found that the hepatoprotective effect of Huangqi Decoction (HQD) on chronic cholestatic liver injury in mice induced by DDC (3,5-diethoxycarbonyl-1,4-dihydrocollidine) may be correlated with the upregulation of Nrf2 and induction of the Nrf2 signaling pathway (Figure 5) (Li et al., 2019). Fragoulis et al. fed wild-type mice, Nrf2 knockout mice, and Keap1 knockout mice with DDC to induce cholangitis. Compared with wild-type and Nrf2 knockout mice, Keap1 knockout mice are almost immune to DDC-induced liver damage. Untreated Keap1 knockout mice have an increased number of intrinsic oogonia compared to wild-type mice, and after DDC treatment, they show stronger oogonia proliferation ability, while Nrf2 knockout mice failed to activate the proliferation of oocytes fully. Therefore, they believe that Nrf2 activation can prevent sclerosing cholangitis and biliary fibrosis caused by DDC (Fragoulis et al., 2019).

### Liver Fibrosis

The activation of hepatic stellate cells (HSC) is a critical reason for the production of extracellular matrix liver fibrosis. Baroni et al. used FeNTA (ferric nitrilotriacetate complex) to stimulate hepatocytes and observed the proliferation of HSC and the accumulation of type I collagen (Svegliati Baroni et al., 1998). It shows that ROS is also one of the activating factors of HSC (Svegliati Baroni et al., 1998). Nrf2 is a transcriptional activator mediated by ROS, promoting the stimulation of a diversity of cytoprotective genes. In Nrf2-deficient mice, the repair of the liver injury that occurred after a single treatment with hepatotoxin carbon tetrachloride (CCl_4_) was distinctly delayed compared to wild-type mice. More importantly, after long-term CCl4 treatment in Nrf2 gene knockout mice, liver fibrosis was severely intensified, and inflammation increased (Xu et al., 2008). Yan et al. found that APAP-induced liver collagen deposition and HSC activation in Nrf2-knockout mice were more severe than wild-type mice (Yan et al., 2018b). They further refined the *in vivo* and *in vitro* experiments and found that andrographolide enhanced nuclear translocation of Nrf2 and increased the expression of antioxidant genes, alleviating APAP-induced liver oxidative stress damage in mice and reducing HSC activation (Yan et al., 2018b). Dwivedi et al. found that DMF can significantly ameliorate the stimulation of HSC induced by thioacetamide, which is related to its suppression of the inflammatory cascade and accumulation of Nrf2 to increase the expression of antioxidants (Figure 5) (Dwivedi et al., 2020). Thus, the induction of Nrf2 is a potential target for treating poison-mediated liver damage and fibrosis.

Liver sinusoidal endothelial cells (LSEC) act a pivotal part in the occurrence and development of liver injury, and their biological signals determine liver regeneration and fibrosis (Ding et al., 2010; Ding et al., 2014). Ruart et al. found that the damage of sinusoidal endothelial cells during acute liver injury can aggravate the oxidative stress response and activate HSC to promote liver fibrosis (Ruart et al., 2019). In the experiment, they also noticed that LSEC with impaired autophagy could not maintain NO levels to maintain its phenotype, leading to the accumulation of ROS and endothelial cell dysfunction (Ruart et al., 2019). The accumulation of ROS and the increase of p62 level caused by impaired autophagy will trigger the stimulation of Nrf2 and the up-regulation of its target gene (Figure 5). However, the up-regulation of Nrf2 mainly depends on oxidative stress, impaired autophagy, or both remains to be elucidated.

### Primary Carcinoma of the Liver

Primary carcinoma of the liver refers to malignant tumors originating from liver cells or intrahepatic bile duct epithelial cells, including HCC, intrahepatic cholangiocarcinoma (ICC) and HCC-ICC mixed type three types Pathological type. Many researchers have found that the expression of cytoprotective genes is an important preventive mechanism for the carcinogenesis of exogenous and endogenous factors. Many natural or synthetic compounds can activate Nrf2 by inactivating Keap1. In non-malignant cells, Nrf2 activation induces the transcription and expression of antioxidant target genes, which can resist genetic damage and physical and chemical carcinogens caused by oxidative stress (Cuadrado et al., 2019). Worldwide, viral hepatitis, especially hepatitis B, is the main cause of HCC (Villanueva, 2019). As mentioned above, Nrf2 is a protective factor for viral hepatitis, but it may contribute to the occurrence and progress of HCC. In addition, the incidence of HCC caused by NAFLD is also gradually increasing (Villanueva, 2019). As mentioned before, the activation of Nrf2 can alleviate NAFLD by regulating liver cell lipid metabolism and oxidative stress. Therefore, it may be a potential preventive measure for NAFLD-related HCC. In East Asia and sub-Saharan Africa, exposure to aflatoxin B1 (AFB1) is another major risk factor besides HBV infection (Forner et al., 2018). Many studies have shown that AFB1 is metabolically activated by hepatocyte CYP450 to form AFBO (aflatoxin B1-8, 9-epoxide) to exert a carcinogenic effect (Rushing and Selim, 2019). Studies have found that in Nrf2 knockout rats, the protective effect of CDDO-Im on AFB1 hepatotoxicity is significantly weakened, which indicates that Nrf2 act a pivotal part in this protection (Taguchi et al., 2016).

However, for the first time, abnormal stimulation of Nrf2 has been observed in non-small cell lung cancer with mutations (Padmanabhan et al., 2006). Subsequently, the researchers identified 226 unique Nrf2 mutant tumors from 10,364 cases in The Cancer Genome Atlas (TCGA). They found that 21 of 33 tumor types have Nrf2 mutations (Kerins and Ooi, 2018). This indicates that such mutations occur frequently. Interestingly, Nrf2 mutations almost appear in ETGE and DLG, but it does not affect the biological activity of Nrf2. As a result, Nrf2 accumulates in the cytoplasm and continues to be transported into the nucleus to activate quantities of target genes. It shows that the loss of protein kinase C (PKC) λ/ι in liver promotes autophagy and oxidative phosphorylation, leading to the generation of ROS (Kudo et al., 2020). The generated ROS drives HCC through Nrf2, which induces antioxidants to maintain ROS below the allowable cell proliferation level without causing cell death (Kudo et al., 2020). Some scholars have found that sorafenib could reduce the production of thioredoxin 1 by down-regulating Nrf2 to reduce liver cancer (González et al., 2020). Therefore, we believe that cancer resistance to chemotherapy and radiotherapy may be related to the detoxification and antioxidant effects caused by the abnormal activation of Nrf2. In addition, high levels of Nrf2 in cells can regulate the metabolism of cancer cells, such as promoting the anabolic pathways of glucose and glutamine, leading to progressive cell proliferation (Mitsuishi et al., 2012). In addition, Liu et al. found that TRIM25 activates Nrf2 through ubiquitination and degradation of Keap1, thereby enhancing hepatocellular carcinoma antioxidant defense capabilities (Liu et al., 2020).

All in all, Nrf2 not only plays an anti-tumor effect, but also plays a tumor-promoting effect, such as promoting cell proliferation and drug resistance.

### Outlook

**FIGURE 6:**
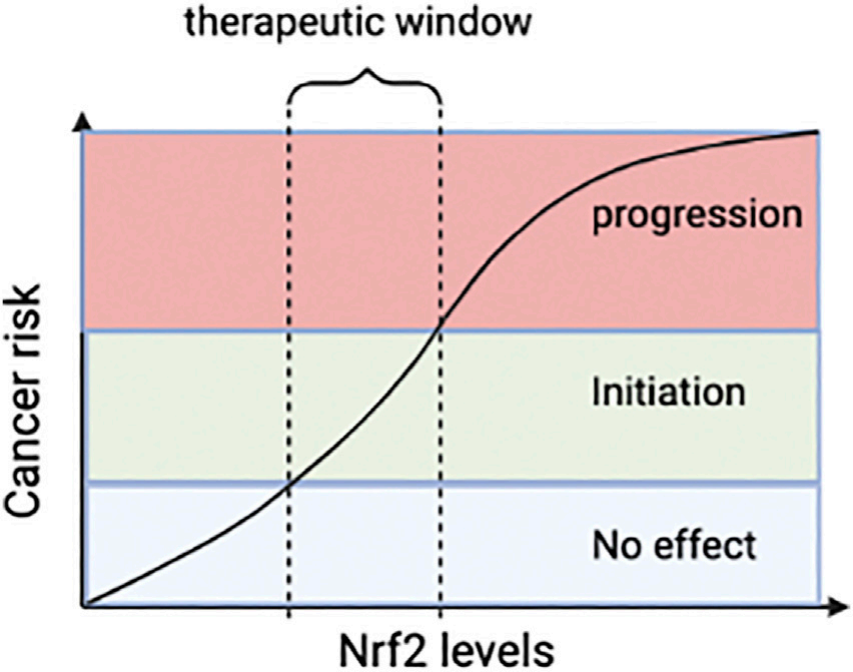
The relationship between Nrf2 and tumor. Reasonable Nrf2 levels may inhibit tumor, but abnormal stimulation of Nrf2 can promote tumor progression. Therefore, dose of Nrf2-drugs should be carefully determined and pharmacological activation of Nr2 for therapy should not exceed the therapeutic window. Nrf2, nuclear factor-erythroid 2-related factor 2.

Oxidative stress is non-disease-specific and a crucial factor in the occurrence and process of many liver diseases. The Keap-Nrf2 pathway is a critical pathway for organisms to resist oxidative stress. The expression of antioxidant protective genes and phase II detoxification enzyme genes induced by it can effectively reduce the sensitivity of liver cells to ROS and electrophiles, slow down the development of liver diseases, and prevent the occurrence and progress of liver fibrosis. However, the current clinical data of Nrf2 agonists are limited and there is no evidence-based basis. Multi-center, large-sample randomized controlled clinical studies are needed for further verification. Furthermore, most of Nrf2 agonists are Keap1 cysteine-targeting compounds. However, they can also interact with other cysteines around the body. It may affect the biological function and bring about drug side effects. Therefore, biopharmaceutical companies should develop drugs that only act on Keap1. Drugs that disrupt the Nrf2-Keap1 protein-protein interactions (PPI) are new targets for treating liver diseases (Figure 2). The advantage of Nrf2-Keap1 PPI inhibitors is improved target selectivity. Due to the extremely short half-life of Nrf2, drugs should be long-lasting, stable and easy to monitor. It is worth noting that the role of Nrf2 in liver cancer has two sides. It may promote tumor cell proliferation and produce drug resistance while anti-tumor. The safe therapeutic window of Nrf2 activators needs to be identified (Figure 6). However, the relevant mechanism is still unclear, and a large number of animal experiments and clinical trials are needed.

## Glossary

AIH: autoimmune hepatitis
AILI: APAP-induced liver injury
ALD: Alcoholic liver disease
APAP: Acetaminophen
ARE: antioxidant responsive element
ASH: alcoholic steatohepatitis
BS: roccoli sprout
BTB: broad complex-tramtrack-bric-a-brac
β-TrCP: β-transducin repeats-containing protein
BVR: biliverdin reductase
bZIP: basic leucine zipper
CCl_4_: Carbon tetrachloride
CMA: chaperone-mediated autophagy
CNC: cap-n-collar
ConA: concanavalin A
CTR: carboxyl terminal region
CUL3: CULLIN3
CYP: cytochrome P450
CYP2E1: cytochrome P450 2E1
DGR: double glycine repeat
DILI: drug-induced liver injury
DMF: dimethyl fumarate
EMA: European Drug Administration
EpRE: electrophile response element
FDA: Food and Drug Administration
GSH: reduced glutathione
GSK-3: glycogen synthase kinase 3
HCC: hepatocellular carcinoma
HO-1: hemeoxygenase-1
HSC: hepatic stellate cell
ICC: intrahepatic cholangiocarcinoma
IVR: intermediate region
Keap1: kelch-like ECH-associated protein 1
LAMP-2A: lysosome-associated membrane protein type 2A
LSEC: liver sinusoidal endothelial cell
MMF: monomethyl fumarate
NAFLD: non-alcoholic fatty liver disease
NAPQI: N-acetyl-1,4-benzoquinone imine
NASH: non-alcoholic steatohepatitis
Neh1: Nrf2-ECH homology domain-1
NQO1: NAD(P)H: quinone oxidoreductase 1
Nrf2: nuclear factor-erythroid 2-related factor 2
PBC: primary biliary cholangitis
PSC: primary sclerosing cholangitis
ROS: reactive oxygen species
SFN: sulforaphane
sMAF: small musculoaponeurotic fibrosarcoma oncogene homologue
SOD: superoxide dismutase
TCM: traditional Chinese medicines
TP: triptolide
UDCA: ursodeoxycholic acid.
